# Synthetic data for privacy-preserving clinical risk prediction

**DOI:** 10.1038/s41598-024-72894-y

**Published:** 2024-10-27

**Authors:** Zhaozhi Qian, Thomas Callender, Bogdan Cebere, Sam M. Janes, Neal Navani, Mihaela van der Schaar

**Affiliations:** 1 University of Cambridge, Cambridge, CB2 1TN UK; 2 University College London, London, WC1E 6BT UK; 3 The Alan Turing Institute, London, NW1 2DB UK

**Keywords:** Synthetic data, Machine learning, Risk-prediction, Outcomes research, Translational research

## Abstract

Synthetic data promise privacy-preserving data sharing for healthcare research and development. Compared with other privacy-enhancing approaches—such as federated learning—analyses performed on synthetic data can be applied downstream without modification, such that synthetic data can act in place of real data for a wide range of use cases. However, the role that synthetic data might play in all aspects of clinical model development remains unknown. In this work, we used state-of-the-art generators explicitly designed for privacy preservation to create a synthetic version of ever-smokers in the UK Biobank before building prognostic models for lung cancer under several data release assumptions. We demonstrate that synthetic data can be effectively used throughout the medical prognostic modeling pipeline even without eventual access to the real data. Furthermore, we show the implications of different data release approaches on how synthetic biobank data could be deployed within the healthcare system.

## Introduction

Medical advances are predicated on the availability of high-quality data, leading to an increasing emphasis on data sharing in both industry and academia. Nevertheless, the sensitivity of medical data is such that it is usually tightly controlled and subject to country-specific legal constraints^1,2^. Consequently, data access remains complex, inconsistent, costly, and time-consuming^3–5^. Synthetic data have been recognized as a promising solution, coupling privacy-preservation with sufficient quality for analysis^6,7^. Generated by algorithm, synthetic data can maintain the statistical properties and distributions of an original dataset but represent newly created participants.

Compared with other privacy enhancing technologies, such as federated learning^8,9^, synthetic data has an unique advantage: all downstream analytical and ML algorithms can be applied to synthetic data in the same way they are applied to real data. The seamless switch between real and synthetic data allows the data user to apply statistical and ML algorithms without replacing or overhauling these tools. Hence, the use cases of synthetic data span the whole life cycle of a data science project, from exploratory data analysis (EDA)^10^ and model development—including dimensionality reduction, cluster analysis, hypothesis testing, feature selection, hyperparameter tuning—through model selection and training.

Two approaches are commonly proposed for deploying synthetic data: “*no-release*” and “*delayed-release*”. Under a *no-release* approach, the data controller only ever releases synthetic data to the user. This allows a variety of applications such as running data science competitions^11^ or the evaluation of new software prior to deployment. Under a *delayed-release* paradigm, the data controller initially makes synthetic data available to a user, followed by the delayed release of the real data. A *delayed-release* approach supports multiple use cases. Users could accelerate projects that may otherwise be delayed as the approval process for real data access is often lengthy, such that many analyses can take months or years to start. Further, the quality and usefulness of any dataset, particularly real-world electronic health records, is often unclear in advance. By using a synthetic version initially, a data user can better understand whether the real data can support the proposed analyses, and identify where there may be issues with the real data that require addressing.

Both of these deployment paradigms require synthetic data that mirrors the conditional distribution between features and outcomes of interest, as well as the relationships between different features. Consequently, any method to generate synthetic data should achieve two goals: imitating the statistical and joint distributions in the real data, and ensuring that the privacy of those present in the original data is preserved. However, these two goals are sometimes in conflict with each other, leading to a trade-off between the usefulness of synthetic data and its privacy^12,13^. At its extreme, a synthetic data generator could memorise an individual’s features and return these in a synthetic dataset^14,15^. Standard generative models are focussed on the first goal of ensuring distributional similarity, while neglecting the second^16,17^. As a remedy, several approaches explicitly designed to allow control over an explicit privacy guarantee have been developed^18–21^. Most commonly, this involves the introduction of noise during the training of a synthetic data generator, such that the generator is presented with a blurred version of reality. However, the additional noise will inevitably perturb the true data distribution, reducing the usefulness of the synthetic data for certain analytical tasks.

Synthetic data have been shown capable of capturing the high-level marginal distributions and pairwise correlations between features^22–26^, as well as in training predictive models^27–30^. However, none of these studies have used synthetic data generators which explicitly control for privacy whilst whether and how synthetic data can be useful in other stages of the data science pipeline is still unexplored. Furthermore, existing studies often use small datasets and idealised prediction tasks for evaluation, raising questions about whether the results extrapolate to more complex and realistic settings^31,32^.

In this study, we aimed to comprehensively examine the utility of synthetic data generated by state-of-the-art privacy-preserving generators at all stages of the clinical risk prediction pipeline. To do this, we created synthetic versions of ever-smokers within the UK Biobank, a large prospective cohort recruited between 2006–2010 with ongoing follow-up through linked records^33^, before developing models to predict 5-year risk of developing lung cancer in both real and synthetic datasets. We show that existing synthetic data generation methods are of sufficient quality to support a broad range of uses under different access paradigms, empowering data controllers to deploy synthetic data for health research and development.

## Results

We used three synthetic data generators: Differentially-Private Generative Adversarial Network (DPGAN)^19^, Private Aggregation of Teacher Ensembles Generative Adversarial Networks (PATEGAN)^20^, and Anonymization through Data Synthesis using Generative Adversarial Networks (ADSGAN)^21^, all of which are specifically designed for privacy-preservation so are suitable for controlled healthcare datasets. DPGAN and PATEGAN are examples of differential privacy-based methods. Differential privacy^34^ is a mathematical framework that ensures the inclusion or exclusion of a single data point does not significantly affect the output of a data analysis, thus providing strong privacy guarantees. On the other hand, ADSGAN is designed based on the privacy notions introduced by the General Data Protection Regulation (GDPR). It is specifically tailored to protect against reidentification attacks, where an adversary attempts to identify individuals within anonymised or pseudonymised datasets. We include both differential privacy-based and non-differential privacy-based methods for completeness, to provide a broad overview of the current landscape in data synthesis approaches.

We also considered PrivBayes^18^, but it did not scale to the size of the dataset. DPGAN, PATEGAN, and ADSGAN are based on generative adversarial networks^35^. This framework involves two opposing models: a generator that creates synthetic participants and a discriminator that attempts to predict whether these synthetic participants were part of the original dataset. Training continues until the data distributions learnt by the generator are indistinguishable from the original dataset. Further details are presented in the Methods. Evaluation of the synthetic datasets with Wasserstein distances, $\alpha$-precision, $\beta$-recall, and authenticity^36^ are presented in Appendix Table 4.

### Exploratory data analysis with synthetic data

#### Descriptive statistics

**Fig. 1:**
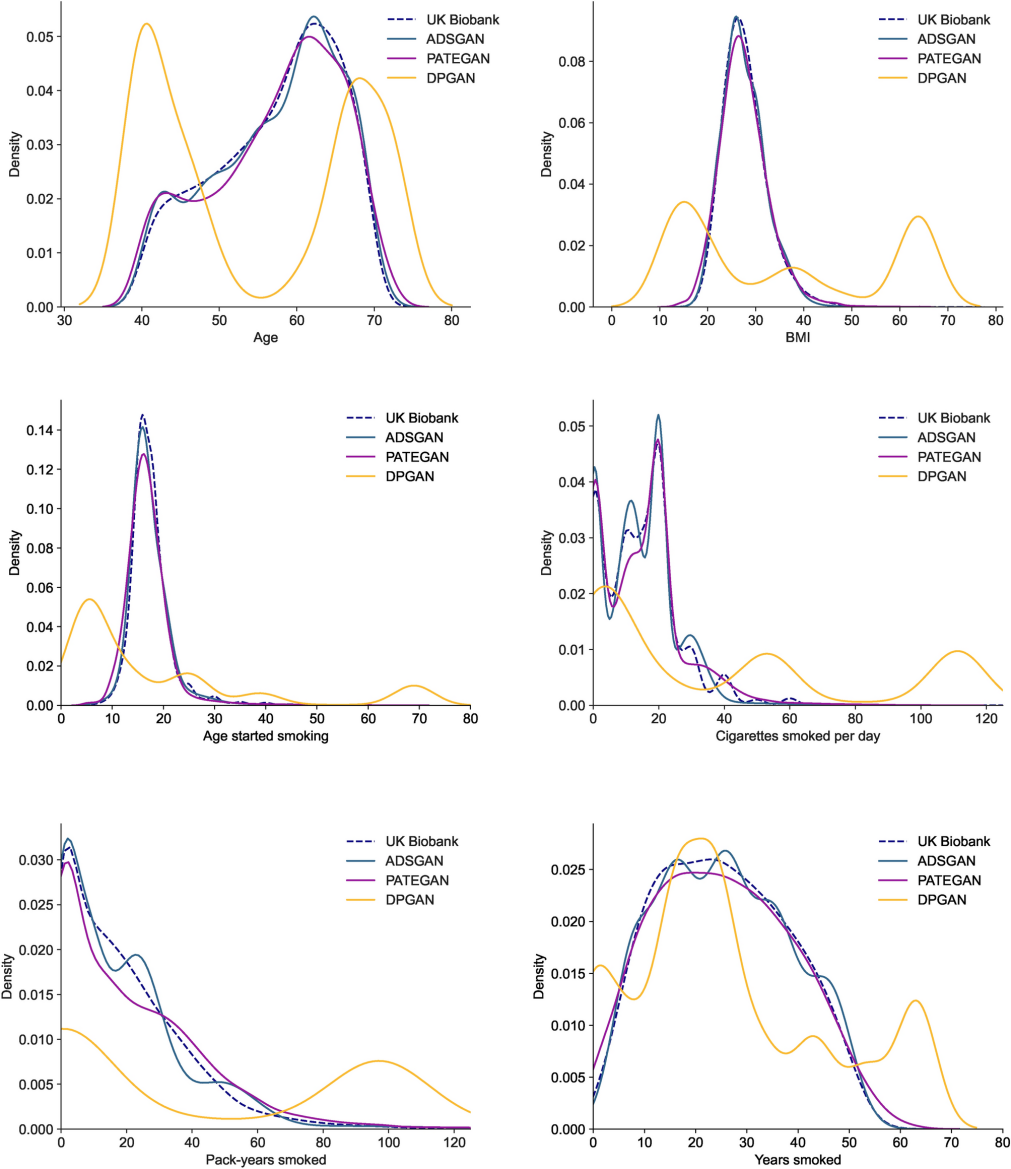
Correspondence between the distribution of continuous variables in the real and synthetic datasets. Synthetic data generated with ADSGAN or PATEGAN maintained the distributions seen in the real training data. DPGAN showed substantial variation from the original and suffered from both mode invention and mode collapse. Abbreviations: ADSGAN, Anonymization through Data Synthesis using Generative Adversarial Networks; PATEGAN, Private Aggregation of Teacher Ensembles Generative Adversarial Networks; DPGAN, Differentially-Private Generative Adversarial Networks.

The descriptive characteristics of the synthetic and real datasets are shown in Table 1. Synthetic datasets generated with ADSGAN and PATEGAN both faithfully represented the training cohort. These methods can also adequately capture the tails of the distribution, including low prevalence conditions (e.g., COPD, Pneumonia, Asthma) and ethnic minority groups. This extended to the complex multi-modal distribution shown in the number of cigarettes smoked per day, where individuals frequently reported values to the nearest five cigarettes (Fig. 1). By contrast, DPGAN struggled to match the distributional characteristics of features, with notable inconsistencies amongst categorical variables, such as an individual’s ethnicity, personal history of cancer, chronic obstructive pulmonary disease (COPD), and pneumonia, along with mode invention and mode collapse amongst key continuous variables. A detailed description of mode invention and collapse and how this occurs when training generative adversarial networks (GANs) is presented in the Appendix.

**Table 1:** Descriptive characteristics of the original and synthetic data.

	Synthetic data			Original data
	ADSGAN	PATEGAN	DPGAN	UK Biobank
Age (mean, SD)	57.41 (8.15)	57.43 (8.34)	54.81 (13.38)	57.39 (7.93)
Sex—Female (n, %)	82,693 (47.70)	86,207 (49.72)	84,264 (48.6)	83,003 (47.88)
Ethnicity—White (n, %)	164,398 (94.82)	165,526 (95.48)	5 (0.0)	166,558 (96.45)
Highest qualification (n, %)				
Degree	48,413 (27.92)	48,038 (27.71)	46,302 (26.71)	47,642 (28.00)
Some college	14,045 (8.10)	12,596 (7.27)	38,521 (22.22)	13,244 (7.78)
Post-secondary school	29,134 (16.8)	27,448 (15.83)	31,442 (18.14)	26,887 (15.80)
Secondary school	46,463 (26.8)	46,228 (26.66)	2,312 (1.33)	46,128 (27.11)
None of the above	35,316 (20.37)	39,061 (22.53)	54,794 (31.61)	36,249 (21.30)
Body mass index (mean, SD)	27.54 (4.57)	27.60 (5.19)	35.86 (21.12)	27.76 (4.78)
Smoking status (n, %)				
Previous	129,883 (74.92)	132,522 (76.44)	49,676 (28.65)	131,822 (76.03)
Current	43,488 (25.08)	40,849 (23.56)	123,695 (71.35)	41,549 (23.97)
Age started smoking (mean, SD)	17.31 (4.34)	16.96 (4.69)	18.39 (19.70)	17.42 (4.33)
Years smoked (mean, SD)	25.64 (12.85)	25.39 (13.51)	26.41 (19.34)	26.32 (12.91)
Cigarettes per day (mean, SD)	14.08 (10.54)	15.04 (12.8)	39.04 (42.59)	18.20 (10.20)
Pack-years (mean, SD)	18.96 (19.52)	21.55 (23.43)	73.86 (76.20)	23.86 (18.90)
Personal history of cancer (n, %)	19,080 (11.01)	17,653 (10.18)	173,368 (99.99)	15,511 (8.95)
COPD (n, %)	7,133 (4.11)	4,872 (2.81)	173,366 (99.99)	5,310 (3.07)
Family history of lung cancer (n, %)	31,495 (18.17)	22,072 (12.73)	173,371 (100.0)	23,144 (13.6)
Pneumonia (n, %)	4,079 (2.35)	3,201 (1.85)	173,367 (99.99)	2,653 (1.53)
Asthma (n, %)	24,780 (14.29)	21,646 (12.49)	172,996 (99.78)	20,464 (11.83)

All datasets included 173,371 participants, equivalent to the size of the real UK Biobank training dataset.

SD, standard deviation; COPD, chronic obstructive pulmonary disease; ADSGAN, anonymization through data synthesis using generative adversarial networks; PATEGAN, private aggregation of teacher ensembles generative adversarial networks; DPGAN, differentially-private generative adversarial networks.

#### Principal component analysis

**Fig. 2:**
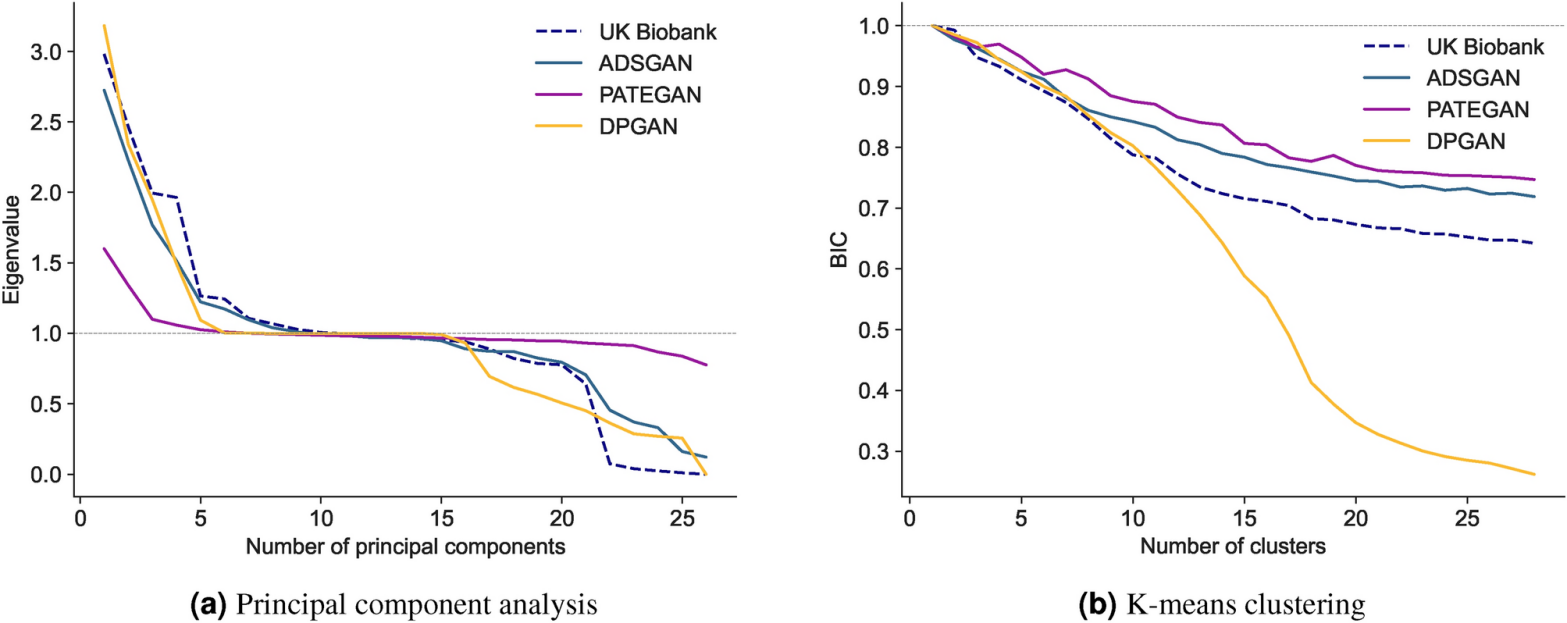
Dimensionality reduction with principal component analysis and K-means clustering in both synthetic and real datasets. Subfigure (**a**) shows the variance explained by different numbers of principal components. Subfigure (**b**) shows the Bayesian Information Criterion (BIC) of K-means clustering with varying numbers of clusters (indexed at one). Abbreviations: ADSGAN, Anonymization through Data Synthesis using Generative Adversarial Networks; PATEGAN, Private Aggregation of Teacher Ensembles Generative Adversarial Networks; DPGAN, Differentially-Private Generative Adversarial Networks.

The first step in principal component analysis (PCA) is to choose the number of principal components, usually by examining the profile of explained variance^37^. As shown in the scree plot in Fig. 2a, the variance explained by number of principal components was similar with ADSGAN and DPGAN to the real data. The profile of PATEGAN was different but an important characteristics was shared: most of the variance was explained by the first four components before the curve flattens out. We subsequently fit PCA models using four principal components on the synthetic datasets before evaluating model quality using the log-likelihood in the real test dataset ($\mathbb {D}^{r}_{test}$). The performance of our PCA model trained on ADSGAN was nearly identical to that trained on the real data $\mathbb {D}^{r}_{train}$, followed closely by PATEGAN (Table 2). Additionally, we show the PCA biplot of the first two principal components generated from the real and different synthetic datasets in Appendix Fig. 3. The analysis above still holds when we study the learned principal components.

#### Clustering with K-means

K-means is a widely used clustering method^38^. Similar to PCA, the number of clusters (*K*) must first be selected. The aim is to find the minimum number of clusters—reducing the dimensions present in the dataset—whilst also reducing the intra-cluster variance. Here we first examine whether synthetic data can help data analysts select the number of clusters. The Bayesian Information Criterion (BIC) is one quantitative metric to guide this choice^39^. Figure 2b shows the BIC profile produced with both real and synthetic datasets with respect to the number of clusters. The overall trends in the BIC were similar across the datasets: the curve decreased until reaching its lowest (best) score at 28 clusters. Note, the BIC with DPGAN paralleled that of the real dataset for 10 clusters before significantly diverging. Although the lowest BIC was at 28 clusters in the real and synthetic data generated by ADSGAN and PATEGAN, the rate of decrease in the BIC reduced significantly after 15 clusters across all three datasets. Therefore, an individual with only synthetic data would still be able to use an analysis of BIC to decide on a reasonable number of clusters.

Subsequently, we performed K-means clustering, with $K = 15$, in both the synthetic and real datasets. We used the clusters identified when training a model with the real training dataset, $\mathbb {D}^{r}_{train}$, as our comparator “oracle”. We show the agreement between the clusters identified in the real data (the “oracle”) and those derived from the synthetic datasets, evaluated on the test set $\mathbb {D}^{r}_{test}$ in terms of the adjusted Rand index (ARI) and adjusted mutual information (AMI) in Table 2. Both metrics would be zero if the clusters were randomly assigned and one if the clustering derived from the synthetic data were in perfect agreement with the oracle. Clusters found from synthetic datasets generated with ADSGAN and PATEGAN agreed well with the oracle. However, the synthetic data generated by DPGAN fell short in producing meaningful clusters.

**Table 2:** Quantitative evaluation results for different analytical tasks.

	PCA	Clustering		Feature selection			Hyperparameters	Model training	
	*l*	ARI	AMI	Precision	Recall	AUROC	Uplift in C-index*	Brier score	C-index
ADSGAN	− 38.430	0.537	0.693	0.615	0.889	0.685	0.017	0.00494	0.698
PATEGAN	− 40.015	0.527	0.697	0.579	0.611	0.644	0.020	0.00504	0.742
DPGAN	− 42.013	0.094	0.130	0.500	0.556	0.463	0.023	0.01969	0.386
“Oracle”	− 38.309	1.000	1.000	1.000	1.000	1.000	0.028	0.00489	0.823

For all metrics, except the Brier score, the larger (or conversely, closer to zero if negative), the better. The “oracle” refers to the models trained on the real training set $\mathbb {D}^{r}_{test}$

PCA, principal component analysis; *l*, log-likelihood; ARI, adjusted rand index; AMI, adjusted mutual information; AUROC, area under the receiver operating curve; C-index, concordance index.

*Relative to a DeepHit model using the default hyperparameters

### Prognostic model development with synthetic data

#### Feature selection

Real world data often contain features that are irrelevant to the prediction task. Here we explored whether feature selection can be reliably performed on synthetic data. The most important features for predicting lung cancer risk in the real data were age, body mass index, smoking duration, pack-years, quit-years, current smoking status, family history of lung cancer and highest qualifications. These features are in keeping with the findings of prior medical literature, with each of the variables included in existing prognostic models for lung cancer^40^.

When performing feature selection with synthetic data, those generated by ADSGAN showed the highest concordance with the real data, keeping all but one of the top ten features: highest qualification (degree). Similarly, feature selection with synthetic data generated by PATEGAN and DPGAN reached similar conclusions, with two discordant features each. The features and associated *p*-values are presented in Appendix Table 5.

To quantify the comparison, Table 2 reports the precision, recall, and AUROC of the true important features when the selection is based on synthetic data. Synthetic data generated by ADSGAN and PATEGAN demonstrated their suitability for feature selection independent of the real dataset, and consistently outperformed those generated by DPGAN.

#### Hyperparameter tuning

Hyperparameter tuning is a complex and important element of prognostic model development and selection. In the slow-release model, hyperparameters can initially be identified using synthetic data and later applied to real data once it becomes available. This approach can significantly save time on the project, if the hyperparameters can be selected from synthetic data reasonably well. To analyse the reliability of hyperparameter tuning with synthetic data, we trained a neural network-based deep survival model, DeepHit, to predict lung cancer occurrence. There are three key parameters—$\alpha$, $\sigma$, and dropout rate—that most significantly impact the performance of DeepHit^41^. By contrast, batch size, hidden dimensionality, learning rate, and patience (for early stopping) are relatively generic deep learning hyperparameters.

The optimal hyperparameters identified when training with real and synthetic datasets were similar across the three key parameters, but with less agreement on the number of hidden dimensions (Table 6). However, this observation is in keeping with prior studies which suggest that the performance of a deep survival model is less sensitive to the number of hidden dimensions^42,43^. We further quantify the usefulness of hyperparameter tuning on synthetic data in Table 2, where we report the improvement in model discrimination (C-index) on the real test dataset $\mathbb {D}^{r}_{test}$. Model tuning in all three synthetic datasets led to a performance gain relative to using the default hyperparameters. These findings suggest that reasonable hyperparameter settings can be identified when using synthetic data that generalize well to real data.

#### Model training

To evaluate the performance of prognostic models trained on synthetic data for real-world deployment without further refitting to real data, we used the train-on-synthetic, test-on-real approach^27^. We developed Cox models using all available features to predict the risk of lung cancer occurrence in each synthetic dataset and in the real training dataset.

Models trained on synthetic data generated by ADSGAN and PATEGAN showed relatively strong discrimination, though less than that achieved when trained on the real data. However, the Brier scores for models trained on ADSGAN-derived synthetic data and real data were equivalent (0.00494 vs 0.00489), with a model trained on PATEGAN-derived synthetic data also performing well. Brier scores quantify the closeness of predicted and observed probabilities and can be decomposed into elements including both calibration and discrimination. This suggests that models trained with ADSGAN and PATEGAN were very well calibrated when tested on real data and able to capture core aspects of the relationship between the variables and the outcome. DPGAN-derived synthetic data were not useful for model development.

Given the trade-off between privacy and utility with synthetic data, a degree of performance drop compared with models trained on real data is unsurprising. However, the strength of the Brier scores suggest that access to synthetic data can inform model development, though further fitting to real data would be necessary to improve their discrimination.

## Discussion

Under real-world data release assumptions, synthetic data generated with existing privacy-preserving algorithms from large scale biobanks can effectively support the development of prognostic models for the 5-year risk of lung cancer. In common with previous analyses, no single generative model was unequivocally best at all tasks. However, in our analyses both ADSGAN and PATEGAN performed consistently well, with limited differences between them across tasks.

We show that synthetic data can be used for exploratory analyses in several ways. First, synthetic datasets adequately preserved the distribution of both continuous and categorical variables from the real dataset in our experiments. Although seemingly straightforward, substantial insight can be derived from descriptive analyses, with uses ranging from project planning and hypothesis generation, to healthcare operations and logistics. Second, by capturing the underlying characteristics and relationships present within the data, synthetic data can be used to select hyperparameters in unsupervised models, shown here by the number of components in Principal Component Analysis and the Bayesian Information Criterion associated with selecting different numbers of clusters. Indeed, we found that both PCA and K-means clustering performed on purely synthetic data translated well to real datasets.

Building on exploratory analyses, we show the value of synthetic data for feature selection, hyperparameter tuning, and model training under the challenging scenario of right-censored time-to-event analyses using both conventional statistical approaches—Cox models—and deep learning. In these analyses, feature selection based on hypothesis testing using synthetic datasets created with ADSGAN and PATEGAN yielded comparable feature sets to the original dataset. Furthermore, the hyperparameters selected for a deep learning model trained on synthetic data were similar to the real dataset, particularly across those hyperparameters such as model $\alpha$, $\sigma$, and dropout rate, that most impact model performance. Finally, Cox models trained on synthetic data had strong Brier scores, approaching that of a model trained on the real dataset, although their discrimination was lower. Given the trade-off between usefulness and privacy, such a drop in performance is expected. Nonetheless, the similarity in Brier scores suggests that synthetic data can be valuable for model development.

Our results have several implications for how synthetic data might be deployed in healthcare settings. Although there are a myriad of different underlying use-cases, how synthetic data could be deployed can largely divided into two approaches: *no-release* or *delayed-release*. Under the most stringent *no-release* situation, the data user has no access to the real data and any analyses they perform on synthetic data will not be validated on the real dataset. Our analyses suggest that synthetic data can still confidently support exploratory data analyses, particularly descriptive analyses, and the planning of further analyses. Nevertheless, the strength of conclusions that can be drawn from prognostic models developed in such a situation will necessarily remain limited. By contrast, multiple use-cases support a *no-release* paradigm where the user has the ability to establish a ground-truth. For example, where the data controller can run code to verify analyses written for synthetic data. In this situation, we show that all aspects of model development could be performed, substantially reducing the risks of data sharing. Furthermore, we also show how synthetic data could support *delayed-release* approaches to data sharing. Through exploratory data analyses and initial model development, a user can ascertain both the suitability of the dataset for the problem they are approaching, and de-risk projects. Subsequently, when the real data become accessible, the user can quickly progress to the application of different modelling approaches.

Synthetic versions of large-scale real-world datasets have been attempted previously for both research-grade primary care data within the UK Clinical Practice Datalink (CPRD)^23^, and the UK National Cancer Registry. However, to date, neither have been able to support use-cases beyond tabulating variable counts, limiting their utility. Consequently, to our knowledge, this is the first work in a clinical context to demonstrate the usability of synthetic data beyond basic descriptive analyses in a complex non-imaging medical dataset.

The practical risks from releasing healthcare datasets are most commonly considered to be from membership inference attacks^44^ and reidentification attacks^45,46^. Membership inference attacks occur when an adversary determines whether a specific individual’s data is present in a dataset. For example, by comparing query results from a machine learning model, an attacker might infer if an individual’s data were included in the training set. Reidentification attacks, on the other hand, often involve the use of auxiliary information to reidentify individuals, even when personal identifiers are removed. For instance, by cross-referencing anonymized genetic data with publicly available demographic databases, it can be possible to uncover the identities of some individuals^47,48^.

Several metrics can be used to evaluate the privacy risks associated with membership inference and reidentification attacks. K-anonymity is a common measure used to assess reidentification risk^49^. It ensures that each individual’s data cannot be distinguished from at least k-1 other individuals’ data in the dataset. More recently, the DOMIAS score^50^ has been developed to evaluate membership inference risk. This score provides a quantitative assessment of the likelihood that an individual’s membership in the dataset can be inferred by an attacker. Both metrics are provided in the Supplementary Materials. These metrics can support data controllers when using tunable privacy-preserving synthetic data generators to decide on the appropriate trade-off between data usability and privacy. Whilst, more generally, such evaluations provide an insight into the risks posed by the release of any synthetic data, whether the data generating mechanism is specifically privacy-preserving or not. Fundamentally, the real world privacy risks will depend on the use case and extent of data release planned.

This work has several limitations. Analyses were performed in one dataset, although the UK Biobank is both large and represents the type of data that is used and shared in a medical context. Further, we curated this dataset and performed imputation prior to synthetic data generation. At present, the generation of high-quality synthetic data in clinical research and development requires such preprocessing. This has advantages in that the data controller will know their data better than any user, but does increase the skillset required by the data controller to generate the synthetic data. Finally, although we show that both ADSGAN and PATEGAN generated high-quality synthetic data, it remains the case that a range of synthetic data generators should be used and evaluated by the data controller before data release. Notably, we found that DPGAN had limited utility. This may reflect the fact that DPGAN is one of the original approaches to integrating differential privacy into synthetic data generation, such that the noise introduced may limit its usefulness at the relatively strong privacy guarantee implied in this analysis.

In summary, synthetic data could be a valuable approach to increasing data sharing, with uses across the whole clinical prognostic modelling pipeline. Whether synthetic data are deployed with or without eventual access to the real data, they can support analyses at all stages from planning to completion, accelerating and de-risking projects, whilst opening new avenues for collaboration and sharing between datasets that have historically remained siloed. Further research to support the deployment of synthetic data in clinical settings should be pursued.

## Methods

### Data and study population

We used data from the UK Biobank, a large prospective cohort of half a million men and women recruited between 2006–10 from across the UK with ongoing follow-up^33^. Lung cancer screening is currently only considered in ever-smokers. Consequently, we included all 216,714 individuals in the UK Biobank without a previous diagnosis of lung cancer at baseline who self-reported as being a current or former smoker. Diagnoses of lung cancer during follow-up were determined through linked national cancer registry data^33^, right censored at 31st July, 2019.

### Variable selection and data pre-processing

We selected 26 candidate variables (Appendix Table 3) either causally linked to lung cancer or used in existing lung cancer prognostic models^40^. To manage missing data, we used multiple imputation with chained equations and predictive mean matching. Prior to analysis, as our synthetic data generators leverage neural networks, we normalised continuous variables such their values lay between 0 and 1. Categorical variables were one-hot encoded.

### Synthetic data generation

For privacy preservation, DPGAN and PATEGAN implement algorithms for differential privacy^34^, whilst ADSGAN is specially designed to protect against re-identification attacks^21^. Differential privacy is a formal, mathematically-definable, notion based on the concept that participation in any database renders a risk of identification, such that it is the relative increase in risk of identification that is of importance^34^. DPGAN achieves differential privacy by adding carefully designed noise to gradients during the learning procedure^51^. Hence, the model weights are updated stochastically rather than based on clean input data only. PATEGAN achieves differential privacy using the Private Aggregation of Teacher Ensembles (PATE) framework, which allows it to tightly bound the influence of any individual sample to the model weights^52^. By contrast, ADSGAN is specially designed to protect against re-identification (linkage) attacks—where publicly available data are combined to re-identify an individual—a type of privacy attack specifically highlighted in the European Union’s General Data Protection Regulation (GDPR)^1^. ADSGAN achieves this by introducing a unique regularization term that encourages generated samples to stay not too close with the real samples.

To train the generators, we split our UK Biobank cohort 80:20 into a training ($\mathbb {D}^{r}_{train}$) and test ($\mathbb {D}^{r}_{test}$) set. As this is a stochastic process, we repeated this ten times using different random seeds. We then generated 10 synthetic datasets, one from each trained generator, and aggregated them into one final synthetic dataset, $\mathbb {D}^{s}$ - a deep generative ensemble^50^. Given randomness in both generators and the synthetic data produced by each generator, deep generative ensembling has been shown to improve the quality of the final synthetic dataset used^50^. This led to three main synthetic datasets, one each for DPGAN, PATEGAN, and ADSGAN. We set a privacy budget of $\epsilon = 1.0$; remaining hyperparameters are available in the Appendix.

### Evaluating synthetic data fidelity

We use Wasserstein distance as an approximation of the distance between probability distributions of real and synthetic data, whilst also reported the Jensen-Shannon divergence commonly used in GANs^53^. Furthermore, we consider the three-dimensional metrics $\alpha$-Precision, $\beta$-Recall, and Authenticity, which characterises the fidelity, diversity and generalization performance of any generative model^54^. We discuss several other related metrics in the Supplementary Material (Appendix Table 4).

### Evaluating synthetic data for exploratory data analysis

We considered the performance of synthetic data for both descriptive analyses and dimensionality reduction. Descriptive analyses were comparative, showing the distributions of both continuous and categorical variables in the synthetic and real datasets. We used kernel density estimation with a Gaussian kernel to produce smoothed plots showing the distribution of continuous variables. For dimensionality reduction, we applied two widely-used techniques: principal component analysis (PCA)^55^ and K-means clustering^38^.

We performed PCA separately on the real training ($\mathbb {D}^{r}_{train}$) and synthetic ($\mathbb {D}^{s}$) datasets, qualitatively comparing the profile of explained variance^37^. The profile of explained variance is an important tool to help decide the number of principal components. Ideally, the PCA model trained on the synthetic $\mathbb {D}^{s}$ should be close to a PCA model trained on the real dataset, $\mathbb {D}^{r}_{train}$, with a similar variance profile. For a quantitative comparison, we also evaluated the two trained PCA models on the real test set, $\mathbb {D}^{r}_{test}$, to measure the difference in their abilities to explain unseen real data in terms of the log-likelihood^56^. We repeated the analysis above for all synthetic data generators.

For K-means clustering, we performed the analysis separately on $\mathbb {D}^{r}_{train}$ and $\mathbb {D}^{s}$ with clusters $k=2, \ldots , 28$. We then qualitatively compared the Bayesian Information Criterion (BIC) curves^57^ obtained from real and synthetic data to evaluate whether synthetic data can help the data user to select the optimal number of clusters. Finally, we applied the trained K-means algorithms to cluster the real test data $\mathbb {D}^{r}_{test}$. We evaluated the agreement in cluster assignment with the adjusted Rand index (ARI)^58^ and adjusted mutual information (AMI)^59^.

### Evaluating synthetic data for model development

We considered two central tasks in model development: feature selection and hyperparameter tuning. For feature selection, we first developed a Cox regression model on the real training data, $\mathbb {D}^{r}_{train}$, to obtain a “ground-truth” p-value, $p_i^{r}$ for each candidate prognostic variable $X_i$, that describes the strength of the association between the variable and lung cancer occurrence. We subsequently repeated this process on each synthetic dataset, $\mathbb {D}^{s}$, obtaining p-values $p_i^{s}$. Keeping those prognostic variables that met a threshold for significance of $\alpha =0.05$, we created lists of selected features from the real and synthetic datasets. By comparing the variables that met this threshold—variables selected in both $\mathbb {D}^{r}_{train}$ and $\mathbb {D}^{s}$ were considered true positives, whilst those selected only in a synthetic dataset were considered false positives—we calculated the precision, recall, and the area under the receiver operating curve (AUROC) of feature selection using hypothesis testing with synthetic data.

For hyperparameter tuning, we trained a deep survival analysis model, DeepHit^41^. We used a randomised search approach for hyperparameter selection, generating a search grid containing 20 different settings. We split our synthetic data, $\mathbb {D}^{s}$, 80:20 into training and validation sets to train and evaluate DeepHit models with different hyperparameters before selecting the best configuration. Finally, we re-trained a model with the selected hyperparameters using the real dataset, $\mathbb {D}^{r}_{train}$ - imitating the delayed-release mode—and evaluated its performance on the real test dataset, $\mathbb {D}^{r}_{test}$, with the concordance index (C-index)^60^. As a baseline, we considered the average performance of the 20 settings on $\mathbb {D}^{r}_{test}$.

### Evaluating synthetic data for model training

To explore the usefulness of synthetic data for model training, we used the train-on-synthetic, test-on-real approach in which we fitted a Cox regression model on the synthetic data, $\mathbb {D}^{s}$, with all candidate prognostic features and then evaluate its performance on the real, test, dataset ($\mathbb {D}^{r}_{test}$). Using the $\mathbb {D}^{r}_{test}$ avoids potential data leakage issues that might occur with an evaluation on the real training datasets, $\mathbb {D}^{r}_{test}$^61^. We considered model discrimination using the concordance index (C-index), as well as model performance and calibration using the Brier score^62^.

## Figures and Tables

**Fig. 3:**
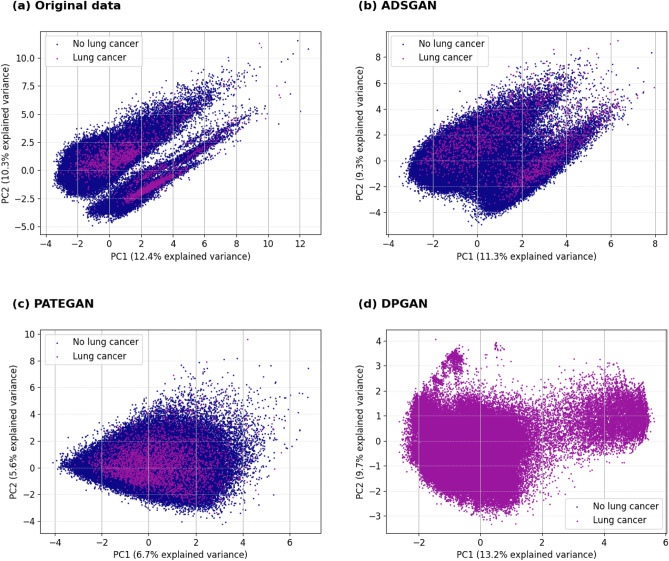
Dimensionality reduction with principal component analysis in both real and synthetic datasets with privacy budget $\epsilon =1$. This figure shows the biplot of the first two principal components with the colors indicating whether the patient has lung cancer. The findings echo those reported in this figure, where the ADSGAN produces PCA nearly identical to the real data, followed by PATEGAN, which captures some aspects of the distribution. DPGAN did not manage to generate meaningful PCA results.

## Data Availability

The code used in this project are available at https://github.com/vanderschaarlab/synthcity. UK Biobank data were used on license (reference 77097) which means that neither the original nor synthetic data can be directly shared. Researchers can apply to the UK Biobank for data access (https://www.ukbiobank.ac.uk/).

## The concept of synthetic data for medical data access

Broadly, synthetic data can be used for data access or data augmentation. Though there are many ways in which datasets can be *augmented* with synthetic data, this is most commonly to manage biases, for example a lack of diversity in a specific dataset, or imbalances in outcomes that make model development challenging. This work focuses on data access.

The use of synthetic data for data access requires several steps: the initial curation of an underlying original, real, dataset that serves to train synthetic data generators; generator training and evaluation; and synthetic data release. A further step is to allow an individual using the synthetic data to run code developed on synthetic data on the original, real, dataset. This is straightforward to facilitate, but requires that the data controller has access to relevant compute—be this locally or through a cloud-based platform—to be able to run the code and return relevant results.

## Understanding mode invention and collapse in generative adversarial networks (GANs)

Generative Adversarial Networks (GANs) are a class of machine learning frameworks designed to generate synthetic data indistinguishable from real data^35^. A GAN consists of two neural networks, a generator and a discriminator, which are trained simultaneously. The generator creates fake data, while the discriminator evaluates their authenticity. This adversarial process aims to improve the generator’s ability to produce realistic data. However, during this training process, phenomena such as mode invention and mode collapse can occur, i.e. invention of non-existing sub populations or eliminating existing sub populations.

Mode invention may introduce artifacts or unrealistic variations that do not correspond to real-world data, potentially misleading downstream applications. Mode collapse limits the effectiveness of the GAN in capturing the complexity of the real data distribution, leading to poor representation of minority sub groups and reduced applicability in medical data synthesis and augmentation.

Various approaches have been adopted to stabilize training and avoid these two issues, including dropout, batch normalization, or gradient penalties. See Tables 3 and 4.

**Table 3:** Candidate variables included in the synthetic data.

Candidate variables	
Age	
Sex	
Body mass index	
Ethnicity	
Highest qualification	
Age started smoking	
Age stopped smoking	
Smoking duration	
Years since stopped smoking	
Pack-years smoked	
Smoking status	
Asbestos exposure	
Personal history of asbestosis	
Personal history of pneumonia	
Personal history of COPD	
Personal history of Emphysema	
Personal history of chronic bronchitis	
Personal history of asthma	
Personal history of eczema, allergic rhinitis, or hayfever	
Personal history of cancer	
Number of previous cancers	
Family history of lung cancer (father)	
Family history of lung cancer (mother)	
Family history of lung cancer (siblings)	

**Table 4:** Evaluating the synthetic datasets.

Metric	ADSGAN	PATEGAN	DPGAN
*Statistical metrics*			
Fidelity ($\alpha$-Precision) $\uparrow$	0.870	0.781	0.177
Diversity ($\beta$-Recall) $\uparrow$	0.301	0.094	0.001
Authenticity $\uparrow$	0.620	0.792	0.954
Wasserstein distance $\downarrow$	0.067	0.292	6.541
Jensen-Shannon distance $\downarrow$	0.003	0.005	0.119
Inverse Kullback-Leibler divergence $\uparrow$	0.993	0.985	0.679
Chi-Squared Test $\uparrow$	0.820	0.875	0.420
Kolmogorov-Smirnov test $\uparrow$	0.941	0.924	0.389
*Privacy metrics*			
k-anonymity $\uparrow$	264	174	999
DOMIAS AUC $\downarrow$	0.384	0.424	0.500

Fidelity refers to $\alpha$-precision; diversity to $\beta$-recall^54^. $\uparrow$ and $\downarrow$ refer to whether a higher or lower value is considered better

**Statistical metrics**

The three-dimensional metrics $\alpha$-Precision, $\beta$-Recall, and Authenticity characterise the fidelity, diversity and generalization performance of any generative model^54^. Statistical distances, such as *Wasserstein distance*, *Jensen-Shannon distance*, and *Kullback-Leibler divergence*, measure the closeness between the real and synthetic data distribution. Statistical tests, such as *Chi-Squared Test* and *Kolmogorov-Smirnov Test*, attempt to test the validity of the null hypothesis that the real and synthetic data are drawn from the same distribution.

**Privacy metrics**

*K-anonymity* is a measure used in data privacy to evaluate the anonymity of a dataset^49^. It ensures that each individual record is indistinguishable from at least $k-1$ other records within the dataset. A higher k-anonymity score indicates better privacy protection, as it means each record is part of a larger group of indistinguishable records.

*DOMIAS* is a density-based Membership Inference Attack (MIA) model that aims to infer membership by targeting local overfitting of the generative model^63^. DOMIAS is shown to be significantly more successful at attacking uncommon samples (e.g. patients with low prevalence conditions and ethnic minority groups). The DOMIAS AUC metric is the AUROC score of an attacker adopting the DOMIAS attack. A higher score indicates more successful attacks.

**Additional metrics for synthetic data Fidelity**.

In addition to the metrics discussed in the main text, several related metrics exist. pMSE score^64^ is defined as $\frac{1}{N} \sum _{i=1}^N (\hat{p}_i - \frac{1}{2})^2$, where $\hat{p}_i$ is the estimated probability that a data point is synthetic, typically obtained by fitting a classifier to distinguish between real and synthetic data. This makes pMSE closely related to the classifier two-sample test methodology^65^. Wasserstein Randomization Test^66^ is a randomization test procedure based on the Wasserstein distance between real and synthetic datasets. We have reported the Wasserstein distance See Tables 5 and 6 and Fig. 3.

**Table 5:** Feature selection with real and synthetic datasets.

Variable	Real	ADSGAN	PATEGAN	DPGAN
Smoking duration (years)	1.3E−58	7.5E−03	9.4E−271	1.2E−32
Age	2.3E−50	1.7E−15	5.3E−159	6.0E−178
Pack-years	5.4E−29	5.1E−20	6.0E−09	0.0E+00
Years since stopped smoking	1.8E−12	1.2E−36	4.2E−09	0.0E+00
Current smoking status	4.6E−11	5.9E−13	8.4E−05	1.3E−83
Family history of lung cancer (father)	1.4E−07	9.2E−05	1.6E−03	–
Family history of lung cancer (siblings)	5.0E−07	8.8E−09	–	–
Highest qualifications—degree	3.0E−05	–	3.2E−08	1.1E−110
Highest qualification—other	2.0E−10	1.9E−04	–	3.1E−03
Body mass index	3.7E−05	3.3E−66	1.5E−98	1.1E−57

*p* values of the top ten features calculated on different data sets. “–” indicates* p* value > 0.05

**Table 6:** Hyperparameter configurations of DeepHit identified from real and synthetic datasets.

	$\alpha$	$\sigma$	Dropout	Batch size	Hidden dim	Learning rate	Patience
Real	0.358	0.358	0.143	200	10	0.001	13
ADSGAN	0.323	0.323	0.129	500	100	0.0001	29
PATEGAN	0.264	0.264	0.106	500	70	0.0001	34
DPGAN	0.463	0.463	0.185	500	90	0.0001	11

## References

[1] European Union. 2018. General data protection regulation (GDPR). https://gdpr.eu/tag/gdpr/. Accessed 22 Nov 2022.

[2] U.S. Department of Health and Human Services - Office for Civil Rights. (2021). Health insurance portability and accountability act of 1996 (HIPAA). https://www.hhs.gov/hipaa/index.html. Accessed 14 Nov 2022.

[3] Blasimme, A., Fadda, M., Schneider, M. & Vayena, E. Data sharing for precision medicine: Policy lessons and future directions. *Health Aff.* **37**, 702–709. 10.1377/hlthaff.2017.1558 (2018). 10.1377/hlthaff.2017.1558 29733719

[4] Ursin, G. et al. Sharing data safely while preserving privacy. *Lancet* **394**, 1902. 10.1016/S0140-6736(19)32603-0 (2019). 31708190 10.1016/S0140-6736(19)32603-0

[5] Mascalzoni, D. et al. Are requirements to deposit data in research repositories compatible with the European union’s general data protection regulation?. *Ann. Intern. Med.* **170**, 332–334. 10.7326/M18-2854 (2019). 30776795 10.7326/M18-2854

[6] Machanavajjhala, A., Kifer, D., Abowd, J., Gehrke, J. & Vilhuber, L. Privacy: Theory meets practice on the map. In *2008 IEEE 24th international conference on data engineering*, 277–286 (IEEE, 2008).

[7] El Emam, K., Mosquera, L. & Hoptroff, R. *Practical synthetic data generation: Balancing privacy and the broad availability of data* (O’Reilly Media, 2020).

[8] Wei, K. et al. Federated learning with differential privacy: Algorithms and performance analysis. *IEEE Trans. Inf. Forensics Secur.* **15**, 3454–3469 (2020).

[9] Mothukuri, V. et al. A survey on security and privacy of federated learning. *Futur. Gener. Comput. Syst.* **115**, 619–640 (2021).

[10] Tukey, J. W. et al. *Exploratory data analysis* Vol. 2 (Reading, 1977).

[11] Jordon, J., Yoon, J. & van der Schaar, M. Measuring the quality of synthetic data for use in competitions. In *KDD Workshop on Machine Learning for Medicine and Healthcare* (2018).

[12] Abowd, J. M. & Vilhuber, L. How protective are synthetic data? In *Privacy in Statistical Databases: UNESCO Chair in Data Privacy International Conference, PSD 2008, Istanbul, Turkey, September 24-26, 2008. Proceedings*, 239–246 (Springer, 2008).

[13] Assefa, S. A. *et al.* Generating synthetic data in finance: Opportunities, challenges and pitfalls. In *Proceedings of the First ACM International Conference on AI in Finance*, 1–8 (2020).

[14] Carlini, N., Liu, C., Erlingsson, Ú., Kos, J. & Song, D. The secret sharer: Evaluating and testing unintended memorization in neural networks. In *USENIX Security Symposium*, vol. 267 (2019).

[15] van den Burg, G. & Williams, C. On memorization in probabilistic deep generative models. *Adv. Neural Inf. Process. Syst.* **34**, 27916–27928 (2021).

[16] Koller, D. & Friedman, N. *Probabilistic graphical models: Principles and techniques* (MIT press, 2009).

[17] Bond-Taylor, S., Leach, A., Long, Y. & Willcocks, C. G. Deep generative modelling: A comparative review of vaes, gans, normalizing flows, energy-based and autoregressive models. In *IEEE transactions on pattern analysis and machine intelligence* (2021). 10.1109/TPAMI.2021.3116668 34591756

[18] Zhang, J., Cormode, G., Procopiuc, C. M., Srivastava, D. & Xiao, X. PrivBayes: Private data release via Bayesian networks. *ACM Trans. Database Syst.* **42**, 1–41. 10.1145/3134428 (2017).

[19] Xie, L., Lin, K., Wang, S., Wang, F. & Zhou, J. Differentially private generative adversarial network. Preprint at arXiv: 1802.06739 (2018).

[20] Yoon, J., Jordon, J. & van der Schaar, M. PATE-GAN: Generating synthetic data with differential privacy guarantees. In *International Conference on Learning Representations* (2019).

[21] Yoon, J., Drumright, L. N. & van der Schaar, M. Anonymization through data synthesis using generative adversarial networks (ADS-GAN). *IEEE J. Biomed. Health Inform.* **24**, 2378–2388. 10.1109/JBHI.2020.2980262 (2020). 32167919 10.1109/JBHI.2020.2980262

[22] Wang, Z., Myles, P. & Tucker, A. Generating and evaluating synthetic UK primary care data: Preserving data utility & patient privacy. In *2019 IEEE 32nd International Symposium on Computer-Based Medical Systems (CBMS)*, 126–131 (IEEE, 2019).

[23] Tucker, A., Wang, Z., Rotalinti, Y. & Myles, P. Generating high-fidelity synthetic patient data for assessing machine learning healthcare software. *NPJ Digit. Med.* **3**, 1–13 (2020). 33299100 10.1038/s41746-020-00353-9 PMC7653933

[24] Goncalves, A. et al. Generation and evaluation of synthetic patient data. *BMC Med. Res. Methodol.* **20**, 1–40 (2020). 10.1186/s12874-020-00977-1 PMC7204018 32381039

[25] Wang, Z., Myles, P. & Tucker, A. Generating and evaluating cross-sectional synthetic electronic healthcare data: Preserving data utility and patient privacy. *Comput. Intell.* **37**, 819–851 (2021).

[26] Kokosi, T. & Harron, K. Synthetic data in medical research. *BMJ Med.* **1**, e000167 (2022). 36936569 10.1136/bmjmed-2022-000167 PMC9951365

[27] Esteban, C., Hyland, S. L. & Rätsch, G. Real-valued (medical) time series generation with recurrent conditional gans. Preprint at arXiv:1706.02633 (2017).

[28] Hittmeir, M., Ekelhart, A. & Mayer, R. On the utility of synthetic data: An empirical evaluation on machine learning tasks. In *Proceedings of the 14th International Conference on Availability, Reliability and Security*, 1–6 (2019).

[29] El Emam, K. Seven ways to evaluate the utility of synthetic data. *IEEE Secur. Priv.* **18**, 56–59 (2020).

[30] James, S., Harbron, C., Branson, J. & Sundler, M. Synthetic data use: Exploring use cases to optimise data utility. *Discov. Artif. Intell.* **1**, 15 (2021).

[31] Pereira, M., Kshirsagar, M., Mukherjee, S., Dodhia, R. & Ferres, J. L. An analysis of the deployment of models trained on private tabular synthetic data: Unexpected surprises. Preprint at arXiv:2106.10241 (2021).

[32] Ganev, G., Oprisanu, B. & De Cristofaro, E. Robin hood and Matthew effects: Differential privacy has disparate impact on synthetic data. In *International Conference on Machine Learning*, 6944–6959 (PMLR, 2022).

[33] Sudlow, C. et al. UK biobank: An open access resource for identifying the causes of a wide range of complex diseases of middle and old age. *PLoS Med.* **12**, e1001779. 10.1371/journal.pmed.1001779 (2015). 25826379 10.1371/journal.pmed.1001779 PMC4380465

[34] *Differential privacy*, vol. 2006 (ICALP, 2006).

[35] Goodfellow, I. et al. Generative adversarial nets. In *Advances in Neural Information Processing Systems* Vol. 27 (eds Ghahramani, Z. et al.) (Curran Associates, Inc., 2014).

[36] Alaa, A., Van Breugel, B., Saveliev, E. S. & van der Schaar, M. How faithful is your synthetic data? Sample-level metrics for evaluating and auditing generative models. In *Proceedings of the 39th International Conference on Machine Learning* Vol. 162 (eds Chaudhuri, K. et al.) 290–306 (PMLR, 2022).

[37] Lorenzo-Seva, U. *How to report the percentage of explained common variance in exploratory factor analysis* (Department of Psychology, 2013).

[38] Arthur, D. & Vassilvitskii, S. K-means++ the advantages of careful seeding. In *Proceedings of the eighteenth annual ACM-SIAM symposium on Discrete algorithms*, 1027–1035 (2007).

[39] Schwarz, G. Estimating the dimension of a model. *Ann. Stat.* **6**, 461–464 (1978).

[40] Toumazis, I., Bastani, M., Han, S. S. & Plevritis, S. K. Risk-Based lung cancer screening: A systematic review. *Lung Cancer* **147**, 154–186. 10.1016/j.lungcan.2020.07.007 (2020). 32721652 10.1016/j.lungcan.2020.07.007

[41] Lee, C., Zame, W., Yoon, J. & van der Schaar, M. DeepHit: A deep learning approach to survival analysis with competing risks. In *AAAI* Vol. 32 10.1609/aaai.v32i1.11842 (2018).

[42] Katzman, J. L. et al. Deepsurv: Personalized treatment recommender system using a cox proportional hazards deep neural network. *BMC Med. Res. Methodol.* **18**, 1–12 (2018). 29482517 10.1186/s12874-018-0482-1 PMC5828433

[43] Nagpal, C., Yadlowsky, S., Rostamzadeh, N. & Heller, K. Deep cox mixtures for survival regression. In *Machine Learning for Healthcare Conference*, 674–708 (PMLR, 2021).

[44] Hu, H. et al. Membership inference attacks on machine learning: A survey. *ACM Comput. Surv. (CSUR)* **54**, 1–37 (2022).

[45] El Emam, K., Jonker, E., Arbuckle, L. & Malin, B. A systematic review of re-identification attacks on health data. *PloS one* **6**, e28071 (2011). 22164229 10.1371/journal.pone.0028071 PMC3229505

[46] Henriksen-Bulmer, J. & Jeary, S. Re-identification attacks-a systematic literature review. *Int. J. Inf. Manag.* **36**, 1184–1192 (2016).

[47] Merener, M. M. Theoretical results on de-anonymization via linkage attacks. *Trans. Data Priv.* **5**, 377–402 (2012).

[48] Harmanci, A. & Gerstein, M. Quantification of private information leakage from phenotype-genotype data: Linking attacks. *Nat. Methods* **13**, 251–256 (2016). 26828419 10.1038/nmeth.3746 PMC4834871

[49] Sweeney, L. Achieving k-anonymity privacy protection using generalization and suppression. *Int. J. Uncertain. Fuzziness Knowl.-Based Syst.* **10**, 571–588 (2002).

[50] van Breugel, B., Qian, Z. & van der Schaar, M. Synthetic data, real errors: How (not) to publish and use synthetic data. In *International Conference on Learning Representations* (2023).

[51] Xie, L., Lin, K., Wang, S., Wang, F. & Zhou, J. Differentially private generative adversarial network. Preprint at arXiv:1802.06739 (2018).

[52] Jordon, J., Yoon, J. & Van Der Schaar, M. Pate-gan: Generating synthetic data with differential privacy guarantees. In *International conference on learning representations* (2018).

[53] Arjovsky, M., Chintala, S. & Bottou, L. Wasserstein generative adversarial networks. In *International conference on machine learning*, 214–223 (PMLR, 2017).

[54] Alaa, A., Van Breugel, B., Saveliev, E. S. & van der Schaar, M. How faithful is your synthetic data? sample-level metrics for evaluating and auditing generative models. In *International Conference on Machine Learning*, 290–306 (PMLR, 2022).

[55] Wold, S., Esbensen, K. & Geladi, P. Principal component analysis. *Chemom. Intell. Lab. Syst.* **2**, 37–52 (1987).

[56] Tipping, M. E. & Bishop, C. M. Mixtures of probabilistic principal component analyzers. *Neural Comput.* **11**, 443–482 (1999). 9950739 10.1162/089976699300016728

[57] Neath, A. A. & Cavanaugh, J. E. The Bayesian information criterion: Background, derivation, and applications. *Wiley Interdiscip. Rev. Comput. Stat.* **4**, 199–203 (2012).

[58] Hubert, L. & Arabie, P. Comparing partitions. *J. Classif.* **2**, 193–218 (1985).

[59] Vinh, N. X., Epps, J. & Bailey, J. Information theoretic measures for clusterings comparison: is a correction for chance necessary? In *Proceedings of the 26th annual international conference on machine learning*, 1073–1080 (2009).

[60] Uno, H., Cai, T., Pencina, M. J., D’Agostino, R. B. & Wei, L.-J. On the C-statistics for evaluating overall adequacy of risk prediction procedures with censored survival data. *Stat. Med.* **30**, 1105–1117 (2011). 21484848 10.1002/sim.4154 PMC3079915

[61] Greener, J. G., Kandathil, S. M., Moffat, L. & Jones, D. T. A guide to machine learning for biologists. *Nat. Rev. Mol. Cell Biol.* **23**, 40–55 (2022). 34518686 10.1038/s41580-021-00407-0

[62] Graf, E., Schmoor, C., Sauerbrei, W. & Schumacher, M. Assessment and comparison of prognostic classification schemes for survival data. *Stat. Med.* **18**, 2529–2545 (1999). 10474158 10.1002/(sici)1097-0258(19990915/30)18:17/18<2529::aid-sim274>3.0.co;2-5

[63] Van Breugel, B., Sun, H., Qian, Z. & van der Schaar, M. Membership inference attacks against synthetic data through overfitting detection. In *Proceedings of the 26th International Conference on Artificial Intelligence and Statistics (AISTATS) 2023* Vol. 162 (PMLR, 2023).

[64] Snoke, J., Raab, G. M., Nowok, B., Dibben, C. & Slavkovic, A. General and specific utility measures for synthetic data. *J. R. Stat. Soc. Ser. A Stat. Soc.* **181**, 663–688 (2018).

[65] Lopez-Paz, D. & Oquab, M. Revisiting classifier two-sample tests. Preprint at arXiv:1610.06545 (2016).

[66] Arnold, C. & Neunhoeffer, M. Really useful synthetic data–a framework to evaluate the quality of differentially private synthetic data. Preprint at arXiv:2004.07740 (2020).

